# A Safe and Efficient 7‐Week Immunotherapy Protocol With Aluminum Hydroxide Adsorbed Bee Venom

**DOI:** 10.1111/all.16528

**Published:** 2025-03-15

**Authors:** Lisa Arzt‐Gradwohl, Urban Čerpes, Eva Schadelbauer, Clemes Schöffl, Sereina Annik Herzog, Christoph Schrautzer, Danijela Bokanovic, Lukas Koch, Karin Laipold, Barbara Binder, Gunter Sturm

**Affiliations:** ^1^ Department of Dermatology and Venereology Medical University of Graz Graz Austria; ^2^ Institute for Medical Informatics, Statistics and Documentation Medical University of Graz Graz Austria; ^3^ Allergy Outpatient Clinic Reumannplatz Vienna Austria

**Keywords:** accelerated up‐dosing protocol, depot bee venom immunotherapy, hymenoptera venom allergy, sting challenge, systemic reaction

To the Editor,

Hymenoptera venom allergy is a potentially life‐threatening disease, and venom immunotherapy (VIT) is the only treatment that can prevent further systemic sting reactions (SSR) and protects 77%–84% of patients treated with honeybee venom and 91%–96% of those treated with vespid venom [1].

Adverse events (AE) are usually rare and mild, and symptoms occur in only 4.3%–11.4% of patients during up‐dosing [2]. A variety of therapy regimes exists for the up‐dosing phase, from conventional to rush and ultrarush or clustered protocols [1]. Current conventional protocols are still time‐consuming for patients. Therefore, we initiated a prospective clinical trial (EudraCT 2015‐002769‐44) evaluating the safety and efficacy of an accelerated up‐dosing protocol with 8 weekly injections in 7 weeks (initial dose of 1 μg followed by 5, 10, 20, 40, 60, 80, and 100 μg, corresponding to 1000, 5000, 10,000, 20,000, 40,000, 60,000, 80,000, and 100,000 SQ at 1‐week intervals by single injections) using the purified depot preparation Alutard SQ bee venom (ALK Abelló, Hørsholm, Denmark). External monitoring was performed during the clinical trial for the purpose of quality assurance.

Seventy‐six patients aged 18–70 years with a history of an SSR to bee stings (≥ grade I, classification of Ring and Messmer [3]) were included (details see Supporting Infromation S1). To demonstrate VIT efficacy, sting challenges with living bees ( *Apis mellifera* ) were performed, whenever possible, already 1 week after reaching the maintenance dose.

Venom immunotherapy was initiated in 75 patients, while it could not be started in one patient due to a medical contraindication. Two patients withdrew from the study at their own request. The remaining 73 patients successfully completed the up‐dosing phase. Seven patients (9.6%, CI 0.00–18.76) showed objective symptoms that were mild to moderate, and two (2.7%, CI 0.00–9.55) additional patients developed subjective systemic reactions (SR; see Tables 1 and 2). Nineteen patients (26.0%) experienced large local reactions (LLR; see Table 1), the majority just once or twice. Elevated (> 11.4 μg/L) baseline tryptase levels (*p* = 0.330), age > 40 years (*p* > 0.999), the prevalence of cardiovascular diseases (*p* = 0.636) or antihypertensive treatment (*p* > 0.999) were not related to the occurrence of SR.

**TABLE 1:** Demographic data and medical history (the numbers and percentages refer to the 75 patients in whom VIT was initiated;) as well as frequency of adverse events (large local and systemic reactions) during the up‐dosing phase and outcome of sting challenges.

Age range (median age) [years]	18–69 (37)
Sex	
Male	33 (44.0%)
Female	42 (56.0%)
Antihypertensive treatment	8 (10.7%)
ACE inhibitor	3 (4.0%)
Beta‐blocker	4 (5.3%)
ACE‐inhibitor and beta‐blocker	1 (1.3%)
Grade of SR (index sting)^a^	
I	10 (13.3%)
II	52 (69.3%)
III	13 (17.3%)
IV	0 (0.0%)
Up‐dosing phase (*n* = 73)	
No side effect	49 (67.1%)
Large local reaction	19 (26.0%)
Objective systemic symptoms	7 (9.6%)
Only subjective systemic symptoms	2 (2.7%)
Sting challenge test (*n* = 71)	
Sting tolerated	56 (78.9%)
Systemic reaction after sting	15 (21.1%)

^a^ According to the classification of Ring & Messmer [3].

**TABLE 2:** Objective, systemic reactions during the up‐dosing phase of venom immunotherapy.

Patient ID	Age	Sex	Reactions (*n*)	Grade^a^	Objective symptoms	Dose of last injection [μg]	Symptoms	Treatment
52	47	Female	2	II; II	No	10; 60	Vertigo; vertigo, globus sensation	Oral antihistamine (second reaction)
72	37	Male	3	I; I; I	No	5; 20; 40	Paresthesia palmar/plantar (all three times)	No
19	39	Female	1	I	Yes	60	Urticaria, pruritus	No
35	59	Male	1	I	Yes	40	Flush	Oral antihistamine
38	31	Female	2	II; II	Yes	1; 5	Vertigo; vertigo, nausea, angioedema	Antihistamine intravenous (second reaction)
47	24	Female	1	II	Yes	40	Abdominal cramping, shivering, cold sweating	Antihistamine (oral and iv.), metamizol‐natrium, butylscopolamin, NaCl
53	48	Female	1	I	Yes	100	Palmar pruritus and erythema	Oral antihistamine
54	31	Female	2	I; I	Yes	60; 100	Palmar pruritus and erythema; palmar pruritus and erythema, urticaria	Oral antihistamine; oral antihistamine and corticosteroid iv.
61	64	Male	1	I	Yes	20	Flush	Oral antihistamine

^a^ According to the classification of Ring and Messmer [3].

Six patients (8.2%) experienced field stings from bees during the up‐dosing phase; one of them developed an exanthema and palmar pruritus 10 min after the sting; all others tolerated the sting. A total of 71 sting challenges were conducted after patients reached the maintenance dose, with 56 patients (78.9%) successfully tolerating the sting. Fifty‐six patients returned to the clinic for the first annual check‐up. Three patients (5.4%) reported a SR after VIT during the first year of the maintenance phase, exhibiting both subjective and objective symptoms. Twenty‐two patients (39.3%) reported field stings, all without any systemic sting reaction.

Adverse events appear to occur less frequently during up‐dosing in conventional protocols compared to faster protocols [1]. However, reaching the maintenance dose in conventional protocols takes a considerable amount of time, leaving patients potentially unprotected for several months. Our objective was to achieve an optimal balance between rapid up‐dosing and safety, with a strong emphasis on the latter. Notably, only 9.6% of patients in our study cohort experienced objective systemic adverse events during the up‐dosing phase.

In the meantime, our published protocol for vespid venom has also been applied to bee venom by another group, though only in 16 patients [4]. Additionally, rush and cluster protocols using depot extracts have been published [5, 6]. A common limitation of all these studies is that their design is underpowered to thoroughly evaluate the safety of bee venom immunotherapy. Moreover, our study is the only one to demonstrate efficacy through controlled sting challenges rather than relying on field sting evaluations. After reaching the maintenance dose of 100 μg, 78.9% of our patients tolerated the sting, aligning with the expected efficacy range of 77%–84%. Our study demonstrated in a substantial cohort of bee venom‐allergic patients that the 7‐week outpatient protocol is safe and effective. Most importantly, it provides patients with faster protection against future systemic sting reactions.

## Author Contributions

**Christoph Schrautzer** and **Gunter Sturm:** conceptualization. **Gunter Sturm:** supervision. **Lisa Arzt‐Gradwohl:** project administration. **Lisa Arzt‐Gradwohl**, **Urban Čerpes**, **Eva Schadelbauer**, **Clemes Schöffl**, **Christoph Schrautzer**, **Danijela Bokanovic**, **Lukas Koch**, **Barbara Binder**, **Gunter Sturm:** investigation. **Sereina Annik Herzog**, **Lisa Arzt‐Gradwohl:** formal analysis. **Sereina Annik Herzog**, **Karin Laipold:** methodology**Lisa Arzt‐Gradwohl** and **Gunter Sturm:** writing‐original draft. **Lisa Arzt‐Gradwohl**, **Urban Čerpes**, **Eva Schadelbauer**, **Clemes Schöffl**, **Sereina Annik Herzog**, **Christoph Schrautzer**, **Danijela Bokanovic**, **Lukas Koch**, **Karin Laipold**, **Barbara Binder**, **Gunter Sturm:** writing – review and editing. The corresponding author attests that all listed authors meet authorship criteria and that no others meeting the criteria have been omitted.

## Conflicts of Interest

Dr. Sturm reports grants from ALK‐Abelló, personal fees from ALK‐Abelló, personal fees from Allergopharma, personal fees from Novartis, and personal fees from Stallergenes‐Greer, outside the submitted work. All other authors have no conflicts of interest to declare.

## Supporting information


Appendix S1.

## Data Availability

The data that support the findings of this study are available from the corresponding author upon reasonable request.
