# Supplementary material for: A Safe and Efficient 7‐Week Immunotherapy Protocol With Aluminum Hydroxide Adsorbed Bee Venom
Source: Allergy. 2025 Mar 15;80(12):3424–6. doi: 10.1111/all.16528 (PMC12666734; doi:10.1111/all.16528)
Supplement: Supplementary file 1 — Appendix S1. [file ALL-80-3424-s001.docx]

**A safe and efficient 7-week immunotherapy protocol with aluminum hydroxide adsorbed bee venom**

The study was approved by the ethics committee of the Medical University of Graz (approval no. 27-405 ex 14/15) and all patients gave their written, informed consent.

**Confirmation of sensitization**Sensitization was confirmed by IgE determination (ImmunoCAP® system, Thermo Fisher Scientific, Waltham, MA, USA), intradermal tests (0.02mL of 0.01, 0.1 and 1 μg/mL) and prick-tests (10, 100, 300 μg/mL solutions). The basophil activation test (Bühlmann Laboratories, Schönenbuch, Switzerland) helped to distinguish between bee and vespid venom allergy in patients with equivocal history and test results. Tryptase levels were determined using the ImmunoCAP® system.

**Venom immunotherapy**

During the up-dosing phase, patients were treated with oral non-sedative antihistamines (histamine (H1) receptor blockers) one hour before injection. The purified depot preparation Alutard SQ*®* bee venom (ALK-Abelló, Hørsholm, Denmark) was administered with an initial dose of 1μg followed by 5, 10, 20, 40, 60, 80 and 100μg corresponding to 1.000, 5.000, 10.000, 20.000, 40.000, 60.000, 80.000, and 100.000 SQ at one-week-intervals by single injections (injection interval: 7 to a maximum of 14 days).

The maintenance phase required single injections every 4-6 weeks with 100μg. All patients were observed for 30 minutes after receiving treatment.
